# A Systematic Review of Artificial Intelligence Applications in the Management of Lung Disorders

**DOI:** 10.7759/cureus.51581

**Published:** 2024-01-03

**Authors:** Akbar Hussain, Stanley Marlowe, Muhammad Ali, Edilfavia Uy, Huzefa Bhopalwala, Dedeepya Gullapalli, Avinash Vangara, Moeez Haroon, Aelia Akbar, Jonathan Piercy

**Affiliations:** 1 Internal Medicine, Appalachian Regional Healthcare, Harlan, USA; 2 Pulmonary and Critical Care, Appalachian Regional Healthcare, Hazard, USA; 3 Diabetes and Endocrinology, Appalachian Regional Healthcare, Whitesburg, USA; 4 Internal Medicine, Appalachian Regional Healthcare, Whitesburg, USA; 5 Cardiovascular, Mayo Clinic, Rochester, USA; 6 Public Health, Appalachian Regional Healthcare, Harlan, USA

**Keywords:** artificial intelligence (ai), lung cancer, machine learning, tuberculosis (tb), chronic obstructive pulmonary disease (copd)

## Abstract

This systematic review examines the transformative impact of artificial intelligence (AI) in managing lung disorders through a comprehensive analysis of articles spanning 2014 to 2023. Evaluating AI's multifaceted roles in radiological imaging, disease burden prediction, detection, diagnosis, and molecular mechanisms, this review presents a critical synthesis of key insights from select articles. The findings underscore AI's significant strides in bolstering diagnostic accuracy, interpreting radiological imaging, predicting disease burdens, and deepening the understanding of tuberculosis (TB), chronic obstructive pulmonary disease (COPD), silicosis, pneumoconiosis, and lung fibrosis. The synthesis positions AI as a revolutionary tool within the healthcare system, offering vital implications for healthcare workers, policymakers, and researchers in comprehending and leveraging AI's pivotal role in lung disease management.

## Introduction and background

Artificial intelligence (AI) has been a revolutionary tool in the past decade, considering its various applications in the diagnosis and management of various diseases. In the past decade, the AI application has gained significant attention in the areas of lung diseases. This introduction lays the foundation for our systematic review of the role of AI in the management of lung disease, and it also establishes a platform for a better understanding of this dynamic intersection [1]. In addition to all these, artificial intelligence algorithms have been utilized in predicting the mortality of critical patients during pandemics such as the SARS-CoV-2 infection, which can have a severe impact on the respiratory system [2].

The prevalence of lung disorders like chronic obstructive pulmonary disease (COPD), pneumoconiosis, and asthma presents persistent challenges to both patients and healthcare systems globally. The advent of AI has introduced transformative possibilities in managing these conditions, leveraging data extraction, imaging advancements, and predictive capabilities. Through our systematic review, we aim to illuminate the evolving landscape of AI in lung disease management, shedding light on its impact and development [1,3]. Our primary focus is to comprehensively explore AI's current role in diagnosing, treating, and preventing lung diseases while critically assessing its strengths and limitations within this crucial domain [3].

## Review

Methods

In conducting this systematic review of Artificial Intelligence (AI) applications in lung disorder management, meticulous adherence to PRISMA guidelines was maintained. The search encompassed the years 2014 to 2023, scouring electronic databases including PLOS ONE, ScienceDirect, PubMed, and Google Scholar and employing a blend of Medical Subject Headings (MeSH) and pertinent keywords such as "lung diseases," "Artificial Intelligence," "Prognosis," "Treatment," and "Diagnosis," in addition, subcategories of AI-machine learning (ML), deep learning, and natural language processing (NLP)- included within our search parameters. The search strategy aimed to yield a broad yet relevant spectrum of literature. A total of 45 studies were initially identified, of which 26 were duplicates. Through a rigorous screening process, 19 studies aligned with the inclusion criteria, focusing on the role of AI in diagnosing and treating lung disorders. These selected studies encompassed various AI modalities, including deep learning, machine learning, and natural language processing, revealing their effectiveness in enhancing diagnostic accuracy and prognostic capabilities across lung diseases such as pneumoconiosis, chronic obstructive pulmonary disease (COPD), tuberculosis, and lung cancer.

**Figure 1:**
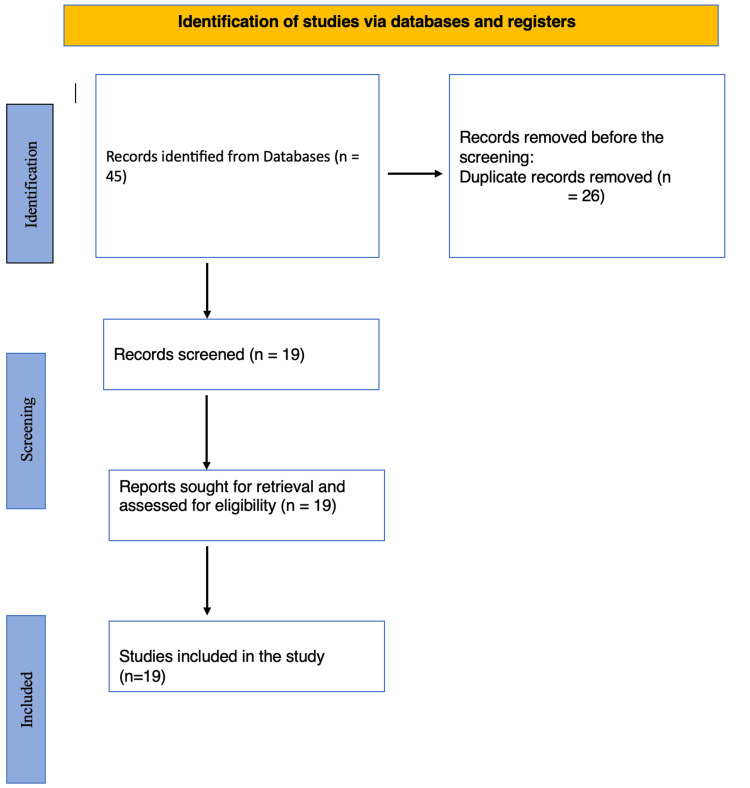
The study selection process summarized by a PRISMA flowchart PRISMA: Preferred reporting items for systematic reviews and meta-analyses

The final review comprised 19 studies, which offered valuable insights into the efficacy of AI tools for managing lung disorders. Specifically, research on pneumoconiosis highlighted the efficiency of deep learning and machine learning models in diagnosis, radiological interpretation, and molecular pathway identification. Studies on respiratory disease detection, including tuberculosis and silicosis, emphasized AI's role in improving diagnostic efficiency in healthcare settings. Furthermore, AI's impact on enhancing lung cancer screening, treatment prediction, and disease recurrence assessment was noted, showcasing its potential for optimizing patient outcomes. Additionally, AI's application in image analysis for diagnosing lung diseases like silicosis underscored its role in automating detection methods and recognizing disease patterns. Molecular mechanism studies elucidated the cellular responses underlying lung fibrosis caused by silica exposure, aiding in targeted therapy development. Moreover, AI models exhibited promise in predicting disease burden and formulating national health programs to mitigate disease impact. A presentation of the summary of the study selection process is shown in Figure 1.

The reviewed studies collectively highlighted AI's strengths in augmenting diagnostic accuracy, efficiency in data analysis, predictive capabilities, and advancements in understanding disease mechanisms. However, the limitations were also acknowledged, including data quality dependencies, interpretability challenges, data privacy concerns, and the need for ongoing research to optimize AI applications fully. In clinical practice, AI's potential was observed in improving diagnostic accuracy, facilitating timely interventions, aiding in research and drug discovery. Future research must focus on standardizing AI models, ensuring interpretability, addressing data privacy, and expanding diverse datasets to bolster robustness and accuracy in lung disease diagnosis. Overall, the integration of AI in healthcare systems has the potential to significantly improve lung disease diagnosis and management by offering personalized and efficient solutions based on individual patient histories and current conditions.

Overview of the included studies

The selected articles explore the applications of AI in the management of lung diseases such as chronic obstructive pulmonary disease and pneumoconiosis. These articles give a better understanding of the implementation of AI in the various aspects of lung disease, including early detection, definitive diagnosis, intervention, disease outcome, and prognosis in the long term.

AI Techniques and Tools Used

A wide variety of AI tools were used in the included studies. Natural language processing (NLP), deep learning, and machine learning were used for data analysis. Trained AI models with knowledge of electronic health records and radiological imaging can significantly improve the efficiency and accuracy of the diagnosis and management of lung diseases such as COPD and pneumoconiosis. Following are the articles that showcased the application of AI in the management of the two most important lung diseases: COPD and pneumoconiosis [1-7].

AI Applications in Pneumoconiosis Diagnosis

Devnath et al. (2022) and Hao et al. (2021) focused on the efficiency of deep learning and machine learning using chest X-rays for the diagnosis of pneumoconiosis. These studies provide a valuable resource for medical professionals to understand the capabilities of deep learning models for better interpretation of the disease [1,3].

Okumura et al. (2017) focused on developing an AI diagnostic system for the detection of pneumoconiosis. They developed an artificial neural network-based system (ANN) and a three-stage ANN method. The ANN method enhanced the classification performance, while the three-stage ANN improved the accuracy. Both of these tools are precious for healthcare professionals [8].

Dong et al. (2022) and Yang et al. (2021) highlight the potential of deep learning in the early detection of diseases, underscoring the importance of imaging analysis to improve accuracy on diagnostic backgrounds [4,5].

Zhang et al. (2021) utilized deep learning techniques for staging, identification, and screening of the signaling pathways in pneumoconiosis. These articles specifically focused on AI implementation at the molecular level [9].

AI Applications in Respiratory Disease Detection

Koul et al. (2023) and Spiegel et al. (2021) focused on the AI role in the identification and diagnosis of tuberculosis, silicosis, and other airway disorders. Both studies' signify AI's importance in healthcare settings for patient care and detection of lung diseases [7,10].

AI Application in Lung Cancer

AI, comprising machine learning and radiomics, revolutionizes lung cancer care by enhancing screening, diagnosis, and treatment. It accurately detects nodules across imaging modalities, improves early detection by utilizing biomarkers, distinguishes benign/malignant nodules, and integrates clinical data to predict treatment responses. AI models optimize therapy selection, showing promise in predicting response and recurrence post-treatment, potentially improving patient outcomes in lung cancer management (Gandhi Z et al.) [11].

AI Applications in Image Analysis for Disease Diagnosis

Zhu (2014), Zhu et al. (2014), and Zhu et al. (2014) utilized AI models such as pattern recognition, radiological image processing, support vector machines, and decision trees. These studies emphasize the automation of detection methods for the early diagnosis of lung diseases such as silicosis. Support vector machines are also significant for the recognition of the pattern of diseases on chest X-rays [12-14].

AI Applications in Molecular Mechanism Understanding

Xiaojun et al. (2016) and Zhang et al. (2021) studied lung fibrosis on the molecular level that is caused by exposure to silica and its cellular response. These studies help in developing targeted therapies for lung diseases [6,9].

AI Applications in Disease Burden Prediction

Lou HR et al. (2022) evaluated the efficiency of the different AI models, such as the ARIMA model, long short-term memory model (LSTM), and deep neural network model (DNN), for the prediction of disease burden. These studies raise awareness regarding public health management based on DALY (disability-adjusted life year) assessments [15].

Rupani et al. (2023) underscore on consequences of silicosis in India. The study indicates the need for a national health program to reduce the disease burden. The programs cover personal protection, health promotion, preventive measures, diagnostic criteria, and symptomatic treatment [16].

Singh (2018) evaluated the utilization of deep learning algorithms for the detection of the disease on chest radiographs. Deep learning is a valuable tool for radiologists for the detection of abnormalities in radiological imaging [17].

Okumura et al. (2011) used a type of AI model, i.e., an artificial neural network upgraded with the spectrum, for the classification of pneumoconiosis on chest X-rays. It helps healthcare professionals in the early detection of lung diseases such as pneumoconiosis on radiological imaging [8] (Table 1).

**Table 1:** AI's role in respiratory disease research: improving diagnosis, detection, and predicting disease burden

No.	Author	Year	Disease	AI Application	AI Tool	Key Findings
1	Devnath et al. [1]	2022	Pneumoconiosis	Diagnosis	Machine Learning	Enhanced interpretability and valuable diagnostic information with deep learning models.
2	Hao et al. [3]	2021	Pneumoconiosis	Diagnosis	Deep Learning	Improved classification performance using an ANN-based system.
3	Dong et al. [5]	2022	Pneumoconiosis	Diagnosis	Deep Learning	Importance of analyzing specific imaging features to improve diagnostic accuracy.
4	Xiaojun et al. [6]	2016	Lung Fibrosis	Molecular Mechanism understanding	-	Contribution to understanding the underlying biology of diseases.
5	Koul et al. [7]	2023	Silicosis, TB, Airway disorders	Detection and Diagnosis	-	Improving the accuracy of diagnostic measures.
6	Okumura et al. [8]	2017	Pneumoconiosis	Diagnosis	Computer Aid Learning (CAD)	A three-stage ANN method for enhanced accuracy and classification of pneumoconiosis.
7	Zhang et al. [9]	2021	Pneumoconiosis	Diagnosis	Deep Learning	Efficient diagnosis and understanding of molecular mechanisms.
8	Spiegel et al. [10]	2021	Silicosis, TB, Airway disorders	Detection and Diagnosis	-	Potential of AI to improve diagnostic efficiency and accuracy in healthcare settings.
9	Zhu and Zhu et al. [12-14]	2014	Pneumoconiosis	Detection and Diagnosis	Image Processing, Pattern Recognition, DTs, SVMs	Automation in image detection processes.
10	Lou et al. [15]	2022	Pneumoconiosis	Disease Burden Prediction	ARIMA model, DNN model, LSTM model	Insights into public health planning and management based on the DALY assessment.
11	Rupani et al. [16]	2023	Silicosis	Disease Burden Prediction		This is a review of the national health program to mitigate the burden of silicosis.
12	Singh et al. [17]	2018		Disease Burden Prediction	Deep Learning	-
13	Carlin et al. [18]	2021	COPD	Detection and Diagnosis	Image Processing	Improves radiological detection of COPD.
14	Exarchos et al. [19]	2021	COPD	Detection and Diagnosis	Deep Learning	Enhance diagnostic accuracy in COPD.

Discussion

The key findings and insight from the articles shed light on the role of AI in the diagnosis, treatment, and prognosis of lung diseases. In the context of pneumoconiosis and chronic obstructive pulmonary disease, AI played a significant role in terms of accurate diagnostic outcomes.

Pneumoconiosis diagnosis: Devnath et al. (2022) and Hao et al. (2021) underscore the efficiency of the deep learning and machine learning models assisting in the diagnosis of pneumoconiosis in radiology. The potential of deep learning techniques is focused on the analysis of radiological imaging features for enhanced accuracy in diagnostic measures [1,3].

COPD diagnosis and management: Exarchos (2022) and Carlin et al. (2021) research on AI's role in COPD is valuable for healthcare professionals and provides a wide range from the diagnosis of the disease to the prediction of the disease outcome and management. These studies provide healthcare workers with a greater understanding to about the AI implementation in various phases of COPD [18,19].

AI techniques and tools: The reviewed studies provided various forms of AI tools such as deep learning, machine learning, ANN, three-staged ANN, natural language processing, etc. These AI tools exploit electronic health records of patients and radiological imaging techniques to improve accuracy in the diagnosis and management of lung diseases [20].

Strengths and Limitations of AI in Lung Diseases

AI in lung disease research has showcased remarkable strengths, notably in enhancing diagnostic capabilities, particularly within radiological imaging. Its adeptness, especially in deep learning models, enables precise identification of subtle changes in images that might elude human interpretation. The efficiency of AI in swiftly processing extensive datasets translates to accelerated diagnostic timelines, offering healthcare professionals accurate insights promptly. Moreover, AI's predictive prowess holds promise for foreseeing disease outcomes, enabling early detection and personalized interventions. Additionally, by elucidating the intricate molecular mechanisms underlying lung diseases like silicosis, AI fosters advancements in research, potentially paving the way for more effective management strategies.

However, AI's application in lung disease research also entails several limitations. Its efficiency heavily relies on the quality and integrity of the data provided. Incomplete or biased datasets can engender AI models prone to biases, potentially resulting in flawed interventions. Furthermore, the opacity of decision-making in certain AI models, particularly deep learning, poses challenges for clinicians in comprehending their predictions fully. Concerns about patient privacy and data security emerge as significant considerations, given the utilization of sensitive medical data in AI applications. Moreover, the dynamic nature of AI in lung disease research necessitates ongoing research to optimize its applications fully and mitigate these limitations.

In clinical practice, leveraging AI offers profound implications for improving diagnostic accuracy, particularly in radiographic assessments. Its efficiency in data analysis facilitates timely interventions, contributing to personalized patient care. Furthermore, by unraveling the molecular intricacies of lung diseases, AI holds potential for steering research towards targeted therapies and drug discovery, promising advancements in treatment modalities. Despite its limitations, the integration of AI stands to significantly transform and enhance clinical approaches to managing lung diseases. Employing simulations and explanatory algorithms enhances transparency and reliability, aiding researchers in choosing the most suitable model for their dataset [21,22]. In the realm of managing lung disorders through AI applications, the increasing use of AI raises ethical and legal complexities. Diverse teams are developing technologies, necessitating international standardization oversight. Acquiring quality data for machine learning encounters hurdles from privacy concerns, regulations, and disparate data systems, hindering standardization and interoperability. Merging data from varied sources poses breach risks, urging researcher immunity for AI medicine progress. To ensure unbiased algorithms, diverse datasets are crucial.

Future research in AI for lung disease management must focus on key objectives: standardization of AI models and algorithms to ensure consistent outcomes and facilitate widespread clinical adoption; development of interpretable AI systems to enhance clinicians’ understanding and trust in AI-generated predictions; addressing data privacy concerns through stringent protocols to build trust among patients and healthcare providers; and expanding diverse datasets to bolster AI model robustness, allowing for better generalization and accuracy in diagnosing lung diseases. These efforts will collectively advance AI’s role, fostering greater reliability, transparency, and efficacy in healthcare settings [23].

## Conclusions

This systematic review sheds light on the emerging importance of artificial intelligence in the healthcare system, especially in the diagnosis and management of various lung diseases, including pneumoconiosis and chronic obstructive pulmonary disease. The reviewed studies centered on exploring the diagnostic potential of AI techniques, including methods like machine learning (ML), deep learning (DL), and natural language processing (NLP). This investigation aimed to examine their accuracy in diagnostic applications. AI models filled with electronic health records and radiological imaging techniques are potential tools for the early detection and prediction of disease outcomes. The practical implications of these key findings in the reviewed articles are significant. The integration of artificial intelligence in the healthcare system can improve the detection methods, prognostic measures, and management plans for diseases. With the assistance of AI models, the diagnosis and management of lung disease can significantly improve. The ability of AI to form personalized health plans is undeniable since it can provide customized solutions based on the patient’s history and current condition.
